# Dental caries in primary and permanent molars in 7-8-year-old schoolchildren evaluated with Caries Assessment Spectrum and Treatment (CAST) index

**DOI:** 10.1186/1472-6831-14-74

**Published:** 2014-06-21

**Authors:** Joanna Baginska, Ewa Rodakowska, Robert Milewski, Anna Kierklo

**Affiliations:** 1 Department of Dentistry Propaedeutics, Medical University of Bialystok, Ul. Waszyngtona 15 a, 15-274 Bialystok, Poland; 2 Department of Restorative Dentistry, Medical University of Bialystok, Bialystok, Poland; 3 Department of Statistics and Medical Informatics, Medical University of Bialystok, Bialystok, Poland

**Keywords:** Caries pattern, CAST index, Children

## Abstract

**Background:**

No reports on a caries pattern covering the full spectrum of the disease could be found in the literature. The aim of this study was to evaluate caries in primary and first permanent molars of 7-8-year-old Polish children by the Caries Assessment Spectrum and Treatment (CAST) index and to find whether there was any correlation between the caries stages in such teeth.

**Methods:**

The study covered 284 7-8-year-old children from randomly selected schools in the Bialystok District, Poland. The prevalence of CAST categories was evaluated with regard to the first and second primary, and first permanent, molars. The Spearman’s rank correlation coefficient was used to explore the correlation of the distribution of CAST codes among the evaluated teeth. The level of statistical significance was established at p < 0.05. The intra-examiner reliability was determined by the unweighted kappa coefficient.

**Results:**

With regard to the permanent molars, caries was observed in 14.8% to 17.3% of the molar and most lesions were scored at the non-cavitation level. Caries in primary molars was most often recorded at the stage of cavitated dentine lesion. Teeth with pulpal involvement, sepsis and extracted due to caries were found to be more prevalent in first, and then in second primary molars. A strong correlation was found between the status of teeth from the right and left sides of the oral cavity. The correlation of the status of first and second primary teeth was stronger for the left than for the right side of the mouth, r was 0.627 and 0.472 in maxilla and 0.513 and 0.483 in mandible (p < 0.001), respectively. For the neighbouring primary and permanent molars the correlation was assessed to be weak. With regard to the teeth situated in opposite jaws the study revealed that the correlations were moderate - r between 0.33 and 0.49. The intra-examiner reliability was established at 0.96 for the primary dentition and at 0.878 for permanent molars.

**Conclusion:**

The strongest correlation found in the evaluated population concerned the distribution of caries in primary molars on the left side of the mouth. The study proved the usefulness of the CAST index in epidemiological surveys.

## Background

Dental caries remains a serious problem in many populations worldwide, with a marked increase of the prevalence in several countries during the last decade [1]. A continued surveillance of the dental epidemiological status is necessary. The Decayed, Missed and Fill teeth (DMFT) index, due to the recommendation of the World Health Organization [2] the most commonly used tool in the epidemiological surveys, has failed to meet the challenges of the 21th century. The detection of pre-cavitated lesions is a matter of importance in the populations with a low prevalence of cavities. The application of the International Caries Detection and Assessment System (ICDAS), in which three stages of enamel lesions are distinguished, may be a solution [3]. However, the system requires using compressed air to dry tooth surfaces and double checking of teeth so that the surveys are costly and time-consuming. For the populations with a high prevalence and a severe course of caries, tools like Pulpal Involvement-Ulceration-Fistula-Abscess (PUFA) and Pulpal Involvement-Roots-Sepsis (PRS) for the detection of consequences of untreated dental caries index were proposed [4,5]. Although PUFA and PRS arouse a great interest, their disadvantage is that they cover only a part of the wide range of caries stages and they only complement the DMFT or ICDAS. From the practical point of view, the most advantageous solution in epidemiological surveys is to use a single index describing the full continuum of a disease.

Recently, an innovative instrument for the epidemiological studies named Caries Assessment Spectrum and Treatment (CAST) was introduced by Frencken at al. [6]. The details concerning the structure of the index were explained in a range of publications [7,8]. A novelty of CAST is the recommendation to include teeth with dental fillings in the category of sound teeth, which is in line with the epidemiological concept of health. A special attention should be given to a modern way of assessing the face and content validity of the instrument by the RAND modified e-Delphi consensus method, with 56 researchers from 24 countries involved in the process [8]. CAST has been already validated in extensive in vitro and in vivo studies which have proven its high specificity, sensitivity and reliability in epidemiological surveys [9,10]. However, the CAST index should be tested in other independent surveys in order to become established as a plausible instrument.

Some universal patterns in caries can be observed, for example: caries levels follow trend lines, there is a specific mathematical relationship between the mean DMFT and mean DMFS, and changes in mean DMFT scores for individuals and groups are not linear [11]. The regularities in caries distribution with regard to teeth, sites and groups of sites were also found [12-16]. The three possible patterns were evaluated: random, aggregated and regular, however, the hypothesis that teeth were randomly infected by caries was rejected. Through the years, the concept of caries regular occurrence, e.g. a symmetrical prevalence with respect to the midline as well as between the upper and lower jaws, was so widely accepted that some measures assessed the level of caries by doubling the results obtained by the examination of a half of the dental arch [11]. However, Vannonberg et al. [16] found that, at the population level, caries had a tendency to symmetrical distribution, but at the individual subject level the cavities rather accumulated on one (left or right) side of the mouth. Batchelor and Sheiham [14] confirmed that a precise symmetry of caries did not occur, but there were groups of teeth with a similar susceptibility. A symmetrical occurrence of lesions resulted in a stepped model of disease levels, i.e. a decline or an increase of caries courses in pairs [11]. The same could be considered for the aggregated pattern. It means that the inclusion of proper preventive measures in the most susceptible groups of sites should result in a substantial caries reduction [14].

Most research studies on the caries pattern were based on a dental evaluation according to DMF [11,12,14,15]. Honcala et al. [17] analyzed the caries distribution and correlation in primary and permanent molar teeth with regard to ICDAS. No reports on a caries pattern covering the full spectrum of the disease could be found in the literature. The aim of this study was to evaluate caries in primary and permanent molars of 7-8-year-old Polish children by the CAST index and to find whether there was any correlation between the caries stages in such teeth.

## Methods

### Study population

The presented data is a part of a cross-sectional survey conducted in the Bialystok District, Poland, between September 2012 and January 2013 under the approval of the Bioethical Committee of the Medical University of Bialystok, Poland (No. R-I-002/352/2012). The study aimed to evaluate the condition of dentition in schoolchildren from randomly selected schools using various caries indices. For the purpose of this manuscript, particularly the data concerning the status of primary and permanent molars in 7-8-year-old children obtained by the CAST index was selected. Parents or caregivers were asked to sign a written statement of consent for child’s participation in the study. In total, 405 children aged between 7 and 8 years were examined during the study. Only those children who had all four permanent molars fully erupted were selected for a further analysis. We also excluded subjects with any of the premolars erupted because in those cases we were not able to determine whether a primary molar was exfoliated or extracted due to caries. Following these criteria we excluded 121 subjects, so the final analysis was performed for 284 children (155 of 7-year-olds and 129 of 8-year-olds). The minimum size of the sample population was calculated on the following assumptions: the number of 7-8-year-old children in this area to be around 9000, the prevalence of caries of deciduous teeth of 80%, a 5% measuring error and a 95% confidence interval. We assumed the percentage of children with caries on the basis of the results from studies previously conducted in this region; in 7-year-old children it reached up to 90% [18-20]. The minimum sample size was determined to be 239 subjects.

### Dental examination

The dental examination was performed by one examiner with ten years of experience in epidemiological surveys. The teeth were evaluated according to the CAST recommendations mentioned in Table 1. The index has a hierarchical structure and covers the full spectrum of caries stages, from a sound surface, pit and fissure sealants, dental fillings, caries lesions in enamel and dentine, a pulpal and periapical inflammation, through to a tooth loss due to caries. The prevalence of particular conditions from tooth reversible premorbidity (enamel lesions) through to tooth’s mortality (extraction) was calculated pursuant to the scheme suggested by Frencken at al. [7]. Prior to the survey, a training session consisting of the theoretical and practical parts was conducted. The theoretical part included the study of the literature and materials provided by the authors of the CAST index; then the extracted primary and permanent molars were evaluated with regard to the presence of CAST codes. The practical part consisted of two sessions of dental examination of 10 children each day.

**Table 1:** Description of CAST codes

**Characteristics**	**Code**	**Description**	**Concept of health**
Sound	0	No visible evidence of a distinct carious lesion is present	Healthy
Sealed	1	Pits and/or fissures are at least partially sealed with a sealant material	
Restored	2	A cavity is restored with a (in)direct restorative material	
Enamel	3	Distinct visual change in enamel only. A clear caries discolouration is visible with or without localised enamel breakdown	Reversible premorbidity
Dentin	4	Internal caries-related discolouration in dentine. The discoloured dentine is visible through enamel which may or may not exhibit a visible localised breakdown of enamel	Morbidity
	5	Distinct cavitation into dentine. The pulp chamber is intact	
Pulp	6	Involvement of pulp chamber. Distinct cavitation reaching the pulp chamber or only root fragments are present	Serious morbidity
Abscess/Fistula	7	A pus containing swelling or a pus releasing sinus tract related to a tooth with pulpal involvement	
Lost	8	The tooth has been removed because of dental caries	Mortality
Other	9	Does not correspond to any of the other categories	

During the survey, the children were examined in school rooms where an artificial light was used to illuminate the oral cavity. All children brushed their teeth before the examination. The status of each tooth surface was checked using a plane dental mirror and a periodontal probe ending with a 0.5 mm ball. The probe was also used for the removal of dental plaque or debris present despite prior tooth-brushing. A dental examination was carried out for all teeth present in the child’s mouth. The status of each tooth’s surface was recorded separately on a form developed for this study. If two conditions were present on the same surface, e.g. a filling in one pit and an enamel lesion in another, or an enamel lesion in one pit and a cavity in another, the higher score was recorded. If an abscess or a fistula was present, all surfaces with an open cavity were scored with code 7. The highest code for each tooth was selected for a further analysis. About 5% of the evaluated population was re-examined at the end of each day in order to determine the intra-examiner reliability.

### Statistical analysis

The prevalence of each caries stage was evaluated with regard to all deciduous and permanent teeth, and separately to the first and second primary, and first permanent, molars. The nonparametric Mann–Whitney U test was used in case of two groups for the comparison of ordinal variables in the statistical analysis. The Spearman’s rank correlation coefficient was used to explore the correlation of the distribution of CAST codes between first and second primary molars, second primary and first permanent molars, the counterpart molar teeth from the right and left side of the dental arch and the molars located in the opposite jaws. The level of statistical significance was established at alfa < 0.05. The fact that, in case of a repeated performance of the test, the alpha level has significantly increased not taking each test separately, but generally all tests together was considered in the statistical analysis. The Bonferroni correction was used in order to prevent it and to maintain the alpha parameter at the level of 0.05, thus to reduce the probability of taking actually random results as significant. The intra-examiner reliability was determined by the unweighted kappa coefficient. The Statistica 10.0 software (StatSoft, Poland) was used for the calculations.

## Results

**Figure 1:**
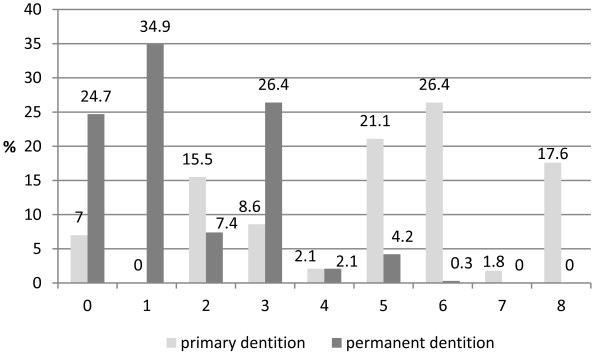
The percentage of children according to the highest CAST category in primary and permanent dentition.

The unweighted kappa value for the intra-examiner reliability was established at 0.96 for the primary dentition and at 0.878 for permanent teeth. Figure 1 shows the percentage of children according to the highest CAST code per mouth, separately for primary and permanent dentition. With regard to deciduous teeth, a quarter of the subjects showed a pulpal involvement (code 6) and one fifth a dentine cavity (code 5) as the most serious caries stages. For permanent teeth, fissure sealants (34.9%) were most prevalent, followed by enamel lesions (26.4%). None of the children scored the categories 7 and 8 in permanent teeth. No tooth scored the category 9 either.

**Figure 2:**
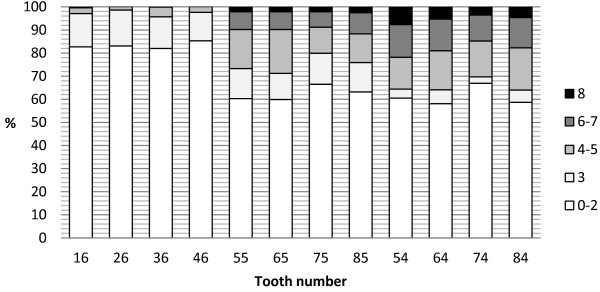
**Distribution of evaluated molar teeth according to the epidemiological concept of health proposed by Frencken et al. **[7]**.**

Table 2 presents the distribution of each CAST code in the evaluated molars. Figure 2 shows the distribution of molar teeth according to different disease stages defined as healthy dentition (codes 0–2), reversible premorbidity stage (code 3), teeth with morbidity (codes 4 and 5) and with serious morbidity (codes 6 and 7), and teeth with mortality (code 8). With regard to the permanent molars, caries was observed in 14.8% to 17.3% of the teeth; however, most lesions were scored at the non-cavitation level (code 3 and 4). About two-thirds of evaluated primary teeth were found to be healthy (codes 0–2). For both, first and second primary molars, caries was most often recorded at the stage of cavitated dentine lesion. A serious morbidity was found to be more prevalent in first, and then in second molars; also extractions were recorded 2–3 times more often in the first than in the second counterparts. The Mann–Whitney U test did not reveal any difference in the distribution of the CAST index in evaluated teeth according to age and sex.

**Table 2:** Distribution of CAST codes in evaluated molar teeth

**Tooth**	**0**	**1**	**2**	**3**	**4**	**5**	**6**	**7**	**8**
**16**	51.4	29.2	2.1	14.4	1.1	1.4	0.4	0	0
**26**	50	29.6	3.5	15.5	0.4	1.1	0	0	0
**36**	39.4	34.9	7.7	13.7	1.4	2.8	0	0	0
**46**	43.7	32.4	9.2	12.3	1.1	1.4	0	0	0
**55**	34.2	0	26.1	13	2.8	14.1	7.7	0	2.1
**65**	36.3	0	23.6	11.3	4.6	14.4	7.7	0	2.1
**75**	38	0.7	27.8	13.4	3.2	8.1	6.7	0	2.1
**85**	33.5	0.7	29.2	12.7	3.2	9.2	9.2	0	2.5
**54**	36.6	0	23.9	3.9	1.1	12.7	13.7	0.4	7.7
**64**	34.9	0	23.2	6	1.1	15.8	12.3	1.4	5.3
**74**	27.8	0	39.1	2.8	1.4	14.1	10.6	0.7	3.5
**84**	26.4	0	32.4	5.3	0.7	17.6	12.7	0.4	4.6

Table 3 shows the results of the repeatedly performed Spearman correlation test where the p-values both without and with the Bonferroni correction are given. The analysis of distribution of the CAST codes in primary second and permanent first molars revealed a strong correlation between caries stages in counterpart teeth from the right and left sides of the oral cavity. Only for the lower first primary molars (84/74) the rank correlation coefficient was lower than 0.5. The correlation of the status of first and second primary teeth was stronger for the left than for the right side of the mouth; r was 0.627 and 0.472 in maxilla and 0.513 and 0.483 in mandible (p < 0.001), respectively. For the neighbouring primary and permanent molars r values were lower than 0.3, which meant a weak correlation. With regard to the teeth situated in opposite jaws the study revealed that the correlations were moderate - r between 0.33 and 0.49. All correlations except the one between second primary and first permanent molars from the left side in maxilla were statistically significant, however, with the Bonferroni correction, the 16/55 correlation was also found to be insignificant.

**Table 3:** The correlations of CAST codes in evaluated molar teeth (Spearman’s correlation coefficient)

	**r**	**p**	p*
Left-right correlations			
16/26	0.513	<0.001	<0.001
46/36	0.638	<0.001	<0.001
55/65	0.501	<0.001	<0.001
85/75	0.594	<0.001	<0.001
54/64	0.611	<0.001	<0.001
84/74	0.495	<0.001	<0.001
Neighbouring teeth correlations			
16/55	0.132	0.025	NS
26/65	0.093	0.114	NS
36/75	0.236	<0.001	<0.001
46/85	0.248	<0.001	<0.001
55/54	0.472	<0.001	<0.001
65/64	0.627	<0.001	<0.001
75/74	0.513	<0.001	<0.001
85/84	0.483	<0.001	<0.001
Upper-lower jaw correlations			
16/46	0.401	<0.001	<0.001
26/36	0.412	<0.001	<0.001
55/85	0.490	<0.001	<0.001
54/84	0.330	<0.001	<0.001
65/75	0.456	<0.001	<0.001
64/74	0.426	<0.001	<0.001

*Bonferroni correction, NS – non-significant.

## Discussion

The contemporary concepts of caries indices are based on the idea of incorporation of all caries stages into one tool. Among many systems, the CAST index stands out with its simple hierarchical structure including the full spectrum of the disease, the categorization of the caries process according to its progression and a modern approach to filled teeth due to their inclusion in the category of sound teeth. CAST is a promising index for epidemiological research studies because the instrument allows obtaining more detailed data on caries prevalence and experience than DMF. Moreover, its use during a survey should be less costly and time-consuming compared to the use of ICDAS, however, such advantages need to be proven in further studies. So far, the reproducibility of CAST in clinical studies was assessed to be from substantial to almost perfect depending on the age of participants [10]. Our study, presenting good intra-examiner reliability in both sets of dentition, proved that CAST could be used in epidemiological studies. The lower kappa value for permanent molars than for primary dentition obtained by us is in accordance with the findings of de Souza et al. [10]. They stated that the level of reproducibility might be influenced by a low prevalence of particular CAST codes, e.g. in the permanent teeth soon after the eruption.

Our findings about a high prevalence of caries, particularly in deciduous teeth, in Polish children are in agreement with the previously reported data [18-21]. 7-year-old children were involved in the Polish National Oral Health Survey in 2011 [20]; 8-year-olds were never a part of such survey. In 2011, only 10.2% of surveyed 7-year-olds in Poland had caries-free primary teeth. The mean d_3_mft was estimated at 4.45 and mean D_3_MFT at 0.42. In our study, the percentage of children found to be caries-free with regard to primary teeth was 7%, however, this proportion would increase to 15.6% if the enamel lesions were excluded from the analysis. For permanent teeth, the percentage of caries-free subjects would be greater for one quarter taking the level of cavitation into dentine as the disease threshold. We observed that the pulpal involvement, the category involving a cavity reaching the pulp or the presence of root fragments, was found to be the most serious stage in 26.4% (primary teeth) and 0.3% (permanent teeth) of the subjects. In 2011, as much as 41.9% of 7-year-old Polish children needed tooth extraction, and 16.4% of them should have had an endodontic treatment. The neglects in dental treatment with regard to the deciduous dentition have been observed worldwide [22-26]. It was previously proven that the dmft level positively correlated with the number of teeth scored with the pufa (pulpal involvement-ulceration-fistula-abscess) index assessing the consequences of untreated dental caries [5].

We decided to primarily concentrate on the correlations between the status of molars because of the considerable dynamics of front teeth exchange in children at the age of 7–8 years. The exclusion of incisors and canines from the analysis allowed us to keep the homogeneity of the study population [27]. A similar approach was adopted by other authors [17,27]. We observed that the percentage of teeth with enamel lesions was at a similar level for second primary and first permanent molars, but with regard to first primary molars the prevalence of code 3 was lower. The tendency that cavitated lesions were more prevalent in primary than in permanent molars was very clear. The presented results are in accordance with the study of Honcala et al. [17] on Estonian children aged 7 and 8 years who assessed molar teeth by the ICDAS criteria. The enamel lesions visible on wet teeth (ICDAS code 2), located on occulsal surfaces of first permanent molars (up to 17% of the teeth) were most prevalent in their study. The highest percentage of teeth with dentine lesions in Estonian children was observed for lower second primary molars. The most probable explanation is that at the age of 7-8-years the factors causing dental caries act too short to induce the development of deep cavities in permanent teeth. Primary teeth are also more prone to a faster lesion progression from enamel to dentine and then to the development of *pulpitis* due to a lower thickness and a relatively larger pulp chamber in comparison to permanent teeth [28]. According to Sheiham and Sabbah [11], the rate of progression of caries through the enamel and into the dentine should determine the frequency of dental recalls.

We found that a large proportion of first and second primary molars with caries lesions at different stages of progression remained untreated. In the present study, the percentage of molars with a serious morbidity (involvement of pulp and tooth surrounding tissues) was especially high for first primary molars, and these teeth also showed the highest tooth mortality (CAST code 8). This observation is in contrast to many previous studies where second primary molars were reported to be more affected than first ones [25,29,30].

The high prevalence of enamel caries in the permanent molars, reaching 15.5% for tooth 36, puts the evaluated Polish children in a group of a high risk of future caries occurrence. The presence of pre-cavitated lesions is a predisposing factor for the cavity development [31]. Masood et al. [32] suggested that obligatory screenings should be started at the age of 6 years and followed for a certain period of time in order to select individuals with the highest caries risk. Another issue increasing the risk of caries in this population is a low percentage of pit and fissure sealants; sealants in permanent teeth were the highest score per mouth for one third of evaluated children (34.9%) only. Occlusal surfaces of permanent molars and buccal pits of lower molars are most prone to the development of caries lesions [14]. Sealants are strongly recommended in the high risk populations [33,34], however, dentists differ in clinical decisions on the indications to seal pits and fissures, on the chosen technique and on the material used as a sealant [35]. The third factor predisposing to caries development in this group is a bad condition of primary teeth. Caries in primary molars is a well known predictive factor for the development of cavities in the permanent dentition, particularly in the first molar teeth [36,37]. Steiner at al. [36] found that a low number of sound primary molars at the age of 7 and 8 years constituted the best and most consistent predictor of a high caries increment in the permanent dentition. According to Gray at al. [36], the presence of three or more deciduous molars at the age of five was the best predictor of caries experience in the first permanent molars at the age of 7 years. Skeie et al. [37] established the level of two surfaces with caries in primary second molars at the age of 5 years as a predictor of caries development during the period of next 5 years.

The present study is one of few reports on the caries pattern distinguishing the disease stages. The symmetry of caries distribution was not previously assessed with regard to the full spectrum of severity of the caries process. We found a strong correlation between the conditions of contralateral molars measured by the CAST index in both, primary and permanent, dentitions. A certain degree of symmetry in caries distribution was also observed for teeth in upper and lower jaws, which is in accordance with the previous reports [11,15]. In our study, the correlations between CAST categories found in neighbouring primary molars were stronger for the left side of the mouth, both in a maxilla and a mandible, which might prove the theory about the accumulative caries pattern [12]. The correlations between caries stages found in first permanent and second primary molars were weak, similarly to the findings of Honcala et al. [17] about the distribution of ICDAS codes on particular surfaces of neighbouring primary and permanent molars. The probable reason is a low number of permanent teeth categorized with CAST codes 5 to 8. A prospective study is needed to assess whether the poor status of deciduous molars influenced the condition of permanent teeth in this population. The lack of such evaluation is undoubtedly one of the limitations of the present report. However, the CAST index is a relatively new research tool and it was impossible to conduct a longitudinal study.

The awareness and understanding of the regularities in the caries pattern should make clinical practitioners more accurate during a dental examination [16]. The practical implication of the described caries pattern is not only that the prevalence of caries lesion in one tooth can be treated as a predictive factor of the caries presence in other teeth from the group of similar susceptibility, but also that a severe caries in one tooth increases the risk of the development of deep cavities and further caries consequences in other teeth.

## Conclusions

The strongest correlation in the evaluated population was found for the distribution of caries stages in primary molars on the left side of the mouth. The study proved the usefulness of the CAST index in epidemiological surveys.

## Competing interest

The authors declare that they have no competing interests.

## Authors’ contributions

JB conceptualised and performed the survey, was involved in the data analysis and the preparation of the manuscript. ER and AK contributed to the data analysis and the preparation of the manuscript. RM performed the statistical analysis and contributed to the preparation of the manuscript. All authors approved the final version of the manuscript.

## Pre-publication history

The pre-publication history for this paper can be accessed here:

http://www.biomedcentral.com/1472-6831/14/74/prepub

## References

[B1] Bagramian RA Garcia-Godoy F Volpe AR The global increase in dental caries. A pending public health crisis Am J Dent 2009 22 3 8 19281105

[B2] World Health Organization Oral health surveys basic methods 1997 4 Geneva: World Health Organization

[B3] Pitts NB Ekstrand KR Intrnational Caries Detection and Assessment System (ICDAS) and its International Caries Classification and Management System (ICCMS) – methods for staging of the caries process and enabling dentist to manage caries Community Dent Oral Epidemiol 2013 41 e41 e52 10.1111/cdoe.12025 24916677

[B4] Monse B Heinrich-Weltzien R Benzian H Holmgren C van Palenstein Helderman W PUFA—an index of clinical consequences of untreated dental caries Commun Dent Oral Epidemiol 2010 38 77 82 10.1111/j.1600-0528.2009.00514.x 20002630

[B5] Baginska J Stokowska W Pulpal Involvement-Roots-Sepsis (PRS) Index: a new method for describing the clinical consequences of untreated dental caries Med Princ Pract 2013 22 555 560 10.1159/000354193 23949116 PMC5586805

[B6] Frencken JE de Amorim RG Faber J Leal S The Caries Assessment Spectrum and Treatment (CAST) index: rational and development Int Dent J 2011 61 117 123 10.1111/j.1875-595X.2011.00022.x 21692781 PMC9374795

[B7] Frencken JE de Souza AL van der Sanden WJM Bronkhorst EM Leal SC The Caries Assessment Spectrum and Treatment (CAST) instrument Community Dent Oral Epidemiol 2013 61 117 123 10.1111/cdoe.12027 24916680

[B8] de Souza AL van der Sanden WJM Leal S Frencken JO The Caries Assessment Spectrum and Treatment (CAST) index: face and content validation Int Dent J 2012 62 270 276 10.1111/j.1875-595X.2012.00121.x 23106841 PMC9374996

[B9] De Souza AL Leal SC Chaves SB Bronkhorst EM Frencken JE Creugers NHJ The Caries Assessment Spectrum and Treatment (CAST) instrument: construct validation Eur J Oral Sci 2014 doi: 10.1111/eos.12116 [Epub ahead of print] 10.1111/eos.12116 24533906

[B10] De Souza AL Bronkhorst EM Creugers NHJ Leal SC Frencken JE The Caries Assessment Spectrum and Treatment (CAST) instrument: its reproducibility in clinical studies Int Dent J 2014 doi: 10.1111/idj.12104 [Epub ahead of print] 10.1111/idj.12104 PMC9376411 24506822

[B11] Sheiham A Sabbah W Using universal patterns of caries for planning and evaluating dental care Caries Res 2010 44 141 150 10.1159/000308091 20389069

[B12] Hujoel PP Lamont RJ DeRouen TA Davis S Leroux BG Within-subject coronal caries distribution patterns: an evaluation of randomness with respect to the midline J Dent Research 1994 73 1575 1580 7929994 10.1177/00220345940730091401

[B13] Mejàre I Stenlund H Caries rates for the mesial surface of the first permanent molar and the distal surface of the second primary molar from 6 to 12 years of age in Sweden Caries Res 2000 34 454 461 10.1159/000016623 11093018

[B14] Batchelor P Sheiham A Grouping of tooth surfaces by susceptibility to caries: a study in 5–16 year-old children BMC Oral Health 2004 4 2 10.1186/1472-6831-4-2 15511295 PMC526778

[B15] Burnside G Pine CM Williamson PR Modelling the bilateral symmetry of caries incidence Caries Res 2008 42 291 296 10.1159/000148161 18663298

[B16] Vanonbbergen J Lesaffre E Garcia-Zattera MJ Jara A Martens L Declerck D Caries patterns in primary dentition in 3-, 5- and 7-year-old children: spatial correlation and preventive consequences Caries Res 2007 41 16 25 10.1159/000096101 17167255

[B17] Honcala E Runnel R Honkala S Olak J Vahlberg T Saag M Mäkinen KK Measuring dental caries in the mixed dentition by ICDAS Int J Dent 2011 2011 50424 doi:10.1155/2011/150424 10.1155/2011/150424 PMC3206401 22114594

[B18] Bagińska J Rodakowska E Wilczyńska-Borawska M Jamiołkowski J Index of clinical consequences of untreated dental caries (pufa) in primary dentition of children from north-east Poland Adv Med Sci 2013 58 442 447 10.2478/v10039-012-0075-x 23793065

[B19] Szafrańska B Waszkiel D Frekfencja i intensywność próchnicy u dzieci w wieku od 3 do 7 lat, mieszkających w Białymstoku Czas Stomatol 2008 61 480 487 [in Polish]

[B20] Wierzbicka M Monitoring Zdrowia Jamy Ustnej. Stan zdrowia jamy ustnej i jego uwarunkowania oraz potrzeby profilaktyczno-lecznicze dzieci w wieku 5,7 i 15 lat 2011 Warszawa: Ministerstwo Zdrowia i Opieki Społecznej [in Polish]

[B21] Emerich K Adamowicz-Klepalska B Dental caries among 7-year-old children in Northern Poland, 1987–2003 Public Health Rep 2007 122 552 558 17639660 10.1177/003335490712200418 PMC1888508

[B22] Honcala E Behbehani JM What’s new in the benefits of restoring primary teeth? Med Princ Pract 2013 22 207 208 10.1159/000348664 23548886 PMC5586754

[B23] Grewal H Verma M Kumar A Prevalence of dental caries and treatment needs in the rural child population of Nainital district, Uttaranchal Indian Soc Pedod Prevent Dent 2009 27 224 226 10.4103/0970-4388.57657 19915273

[B24] Matulaitiene ZK Zemaitiene M Zemgulyte S Milciuviene S Changes in dental caries and oral hygiene among 7-8-year-old schoolchildren in different regions of Lithuania 1983–2009 Stomatologija Baltic Dental and Maxillofacial J 2012 14 53 59 59 23041912

[B25] Zhang S Liu J Lo ECM Chu C-H Dental caries status of Dai preschool children in Yunnan Provence China. BMC Oral Health 2014 14 16 10.1186/1472-6831-14-16 PMC4222259 24279504

[B26] Zhang S Liu J Lo ECM Chu C-H Dental caries status of Bulang preschool children Southwest China BMC Oral Health 2013 13 68 10.1186/1472-6831-13-68 24593701 PMC3946148

[B27] Stephenson J A model for the analysis of caries occurrence in primary molar tooth surface Caries Res 2012 46 452 459 10.1159/000339390 22739707

[B28] Lynch RJM The primary and mixed dentition, post-eruptive enamel maturation and dental caries: a review Int Dent J 2013 63 Suppl 2 3 13 24283279 10.1111/idj.12074 PMC9375027

[B29] Elfrink MEC Veerkamp JSJ Kalsbeek H Caries pattern in primary molars in Dutch 5-year-old children Eur Arch Paediatr Dent 2006 7 236 240 10.1007/BF03262558 17164068

[B30] Wyne AH Caries prevalence, severity and pattern in preschool children J Contemp Dent Pract 2008 9 24 31 18335116

[B31] Steiner M Helfenstein U Marthaler TM Dental predictors of high caries increment in children J Dent Res 1992 71 1926 1933 10.1177/00220345920710121401 1452896

[B32] Masood M Yusof N Hassan MI Jaafar N Assessment of dental caries predictors in 6-year-old school children – results from 5-year retrospective cohort study BMC Oral Health 2012 12 989 10.1186/1471-2458-12-989 PMC3524020 23158416

[B33] Berger S Goddon I Chen C-M Senkel H Hickel R Stösser L Heinrich-Weltzien R Kühnisch J Are pit and fissure sealant with a higher caries risk? Clin Oral Invest 2010 14 613 620 10.1007/s00784-009-0343-8 19798521

[B34] Oulis CJ Berdouses ED Mamai-Homata E Polychronoulou A Prevelance of sealants in relation to dental caries on the permanent molars of 12 and 15-year-old Greek adolescents A national pathfinder survey. BMC Oral Health 2011 11 100 10.1186/1471-2458-11-100 PMC3048527 21320343

[B35] Courson F Velly AM Droz D Lupi-Pégurier L Muller-Bolla M Clinical decision on pit and fissure sealing according to the occlusal morphology. A descriptive study Eur J Paediatr Dent 2011 12 43 49 21434735

[B36] Gray MM Marchment MD Anderson RJ The relationship between caries experience in the deciduous molars at 5 years and in first permanent molars of the same child at 7 years Community Dent Health 1991 8 3 7 2049653

[B37] Skeie MS Raadal M Strand GV Espelid I The relationship between caries in the primary dentition at 5 years of age and permanent dentition at 10 years of age - a longitudinal study Int J Paediatr Dent 2006 16 152 160 10.1111/j.1365-263X.2006.00720.x 16643535

